# Severe euglycemic ketoacidosis following combined therapy with GLP-1 receptor agonist and SGLT-2 inhibitor, refractory to standard treatment: a case report

**DOI:** 10.3389/fendo.2025.1649270

**Published:** 2025-10-07

**Authors:** Agnieszka Gandecka-Pempera, Dariusz Naskręt, Anna Adamska, Dorota Zozulińska-Ziółkiewicz

**Affiliations:** Department of Internal Medicine and Diabetology, Poznan University of Medical Sciences, Poznan, Poland

**Keywords:** euglycemic diabetic ketoacidosis (euDKA), SGLT-2 inhibitor, GLP-1 receptor agonist, type 2 diabetes mellitus, adverse drug reaction

## Abstract

**Background:**

Euglycemic diabetic ketoacidosis (euDKA) is a rare but potentially life-threatening complication of diabetes, increasingly linked to SGLT-2 inhibitors (SGLT-2i) and, less frequently, GLP-1 receptor agonists (GLP-1 RA), especially in combination.

**Case presentation:**

We present the case of a 36-year-old man with newly diagnosed type 2 diabetes who developed severe euDKA within days of starting combined therapy with semaglutide and dapagliflozin. Despite prior patient education and adherence to antidiabetic therapy, persistent vomiting and carbohydrate deficiency—likely related to GLP-1 RA side effects—led to decreased insulin administration and metabolic decompensation. This case was characterized by very early onset after initiation of therapy, absence of infection or other common triggers, and resistance to standard treatment with insulin, glucose, and fluid resuscitation. Intensive care with continuous veno-venous hemodiafiltration was required, resulting in full recovery.

**Conclusion:**

This case emphasizes the potential severity of euDKA induced by GLP-1 RA and SGLT-2i combination therapy and highlights the risk of treatment resistance. The decision to initiate SGLT2i and GLP-1RA concurrently should be individualized or possibly spread apart in time, weighing the potential therapeutic benefits against the risk of rare but severe complication such as euDKA. Comprehensive education on ketone monitoring and insulin administration during intercurrent illness are essential to prevent this rare but serious complication.

## Introduction

Diabetic ketoacidosis (DKA) is a severe, life-threatening acute complication of diabetes. It occurs more frequently in individuals with type 1 diabetes; however, it may also affect patients with type 2 diabetes (1). DKA is primarily caused by insulin deficiency, as seen in new-onset diabetes or omission of insulin therapy. It may also result from increased insulin demand during conditions such as infections or acute medical events (eg. stroke, myocardial infarction). It is characterized by hyperglycemia, the presence of glucosuria and ketonuria, ketonemia and corresponding alterations in arterial blood gas analysis indicative of metabolic acidosis (1, 2). Clinically, DKA presents with nausea, vomiting, rapid and deep breathing (Kussmaul respiration) and significant dehydration. Euglycemic diabetic ketoacidosis (euDKA) is a relatively rare variant of DKA, with an incidence ranging from approximately 2.6-3.2% (3). The clinical manifestation is similar; however, the distinguishing feature is the absence of significant hyperglycemia, with blood glucose levels remaining below 200 mg/dl (11.1 mmol/l) (4–8). Possible causes of euDKA include prolonged fasting or starvation, persistent vomiting, pancreatitis, pregnancy, sepsis, excessive alcohol consumption and intake of certain medications (9). Mortality in euDKA may be comparable to, or even higher than, that in classical DKA due to the potential for delayed diagnosis and treatment resulting from the absence of marked hyperglycemia (10). According to an analysis of the FDA Adverse Event Reporting System (FEARS) database, the mortality rate for euDKA associated with sodium-glucose transporter-2 inhibitors (SGLT-2i) use was approximately 2.5% (11), which is slightly higher than the mortality rate observed in classical DKA, estimated at around 1% (1). Drug-induced euDKA is most commonly associated with the use of SGLT-2i in patients with insulin deficiency (12). However, cases related to glucagon-like peptide-1 receptor agonists (GLP-1 RA) - have also been reported, particularly in the presence of additional risk factors such as a low-carbohydrate diet, nausea and vomiting leading to dehydration, gastrointestinal infections, or acute pancreatitis (13, 14). Giving the increasing use of GLP-1 RA and SGLT-2i, as well as their combination therapy, it is important to remain aware of the potential risk of euDKA and to monitor patients for its symptoms even in the presence of normal blood glucose levels.

## Case presentation

The patient, 36-year old man with new onset of diabetes mellitus, was initially admitted to the Department of Diabetology due to markedly elevated blood glucose levels (277 mg/dl, 15.4 mmol/l), glucosuria, trace amounts of acetone in the urine and with normal arterial blood gas findings. Medical history revealed a 6-month period of polydipsia, polyuria, and a weight loss of approximately 15 kg in 2 years. He had been diagnosed with prediabetes for approximately 8 years and was previously treated with metformin. His initial body weight was 119 kg at a height of 191 cm (BMI 32,6 kg/m2). Glycated hemoglobin A_1c_ (HbA_1C_) at the onset of diabetes was 11.6% (102.6 mmol/mol). Autoantibody testing for anti-GAD, anti-ICA, and anti-IA2 antibodies was negative. Hypertension and hypertriglyceridemia were diagnosed. Abdominal ultrasound revealed hepatic steatosis. Proteinuria was detected despite a normal serum creatinine level. Treatment was initiated with a basal-bolus insulin regimen using functional insulin therapy, combining NPH insulin with insulin lispro. Metformin was continued and semaglutide at a dose 0.25 mg once a week and dapagliflozin 10 mg daily were added. Subsequently, basal NPH insulin was discontinued due to good glycemic control. At discharge, the patient was advised to continue prandial insulin lispro and to monitor urinary ketones in the event of symptoms such as nausea and vomiting or adherence to a carbohydrate-free diet.

On the evening following hospital discharge, two days after the first dose of semaglutide, the patient developed nausea and severe vomiting. He attributed these symptoms to possible food poisoning or a gastrointestinal infection. Due to persistent vomiting, he was unable to eat, so consequently, he did not administer prandial insulin, only occasionally using small doses of rapid-acting insulin once or twice daily in response to elevated blood glucose levels. He continued oral medications, including the SGLT-2i. On the sixth day of persistent vomiting, due to significant dehydration and weakness, the patient was admitted to the emergency department of a hospital, where severe diabetic ketoacidosis was diagnosed. He was subsequently transferred to the Diabetology Department. On initial assessment, the patient’s general condition was severe. He was conscious, afebrile but dehydrated with a heart rate of 105 bpm, blood pressure 110/60 mmHg and exhibited Kussmaul respiration. His body weight was 110 kg, reflecting an additional weight loss of 8 kg since the previous hospitalization. Plasma blood glucose was 182 mg/dl (10.1 mmol/l). Venous blood gas assessment confirmed severe metabolic acidosis, with pH 7.066, pCO_2_ 10.1 mmHg, HCO_3–_7 mmol/l, BE -26,1 mmol/l.Capillary ketones (beta-hydroxy butyrate) were 5.1 mmol/l, urinalysis revealed ketonuria and glucosuria. Serum creatinine was within normal limits, as were C-reactive protein and liver function tests. A diagnosis of euDKA was made. Other etiologies, such as metformin-induced lactic acidosis and infection, were ruled out with additional testing (a lactic acid level of 1 mmol/l, no signs of acute pancreatitis in CT scan, no signs of pneumonia in a chest X-ray).

### Treatment

Upon admission, treatment was initiated with an intravenous infusion of 0,9% saline with potassium, as well as continuous intravenous insulin, in accordance with the DKA management protocol of the Polish Diabetes Association (15). Additionally, due to blood glucose levels below 200 mg/dl (11.1 mmol/l), a 5% glucose and follow 10% glucose intravenous infusion was initiated from the beginning of treatment. Despite standard treatment, the patient’s clinical condition and blood gas parameters did not improve over the following hours. After 24 hours of treatment, the pH level was 7.09, pCO_2_ 6.8 mmHg, HCO_3_- 6.6 mmol/l, BE -27.1 mmol/l. Due to the lack of improvement in laboratory results, the administration of bicarbonates was considered. However, given the patient’s rapidly deteriorating general condition, altered consciousness, tachypnea, and respiratory muscle fatigue, a decision was made to transfer him to the intensive care unit, where continuous veno-venous hemodiafiltration (CVVHDF) therapy was immediately initiated and continued for 48 hours with gradual resolution of acidosis symptoms and normalization of blood gas parameters. Upon resumption of oral nutrition, a multiple daily injection (MDI) insulin regimen was initiated using a combination of basal insulin glargine and prandial insulin lispro. The remainder of hospitalization was uneventful and the patient was ultimately discharged home with recommendations to continue treatment using MDI insulin regimen, without any oral hypoglycemic agents. GLP-1RA and SGLT2i therapy was also not resumed. The laboratory test results during the course of the case are presented in **Table 1**.

**Table 1:** Patient characteristics across different treatment phases.

Clinical and laboratory parameters	At diabetes diagnosis	At hospital admission with euDKA	After 24 hours of treatment with standard protocol	After 48 hours of CVVHDF treatment in ICU	At hospital discharge
HbA_1c_ (%) (mmol/mol)	11.6 102.6				
Body weight (kg)	119	110			105
BMI (kg/m^2^)	32.6	30.1			28.7
Glucose level (mmol)	15.4	10.1	10.3	8	
Creatinine level (mg/dl) (μmol/l)	0.68 60.1	0.89 78.7	0.57 50.4		0.61 53.9
CRP (mg/l)	6.07	4.25	13.6	64	6.55
Presence of glucose (mg/dl) and ketones in urine sample	Glucose 2000 mg/dl, Ketones (+)	Glucose 2000 mg/dl, Ketones (+++)		Glucose 2000 mg/dl, Ketones (++)	Glucose 2000 mg/dl, Ketones (-)
pH	7.418	7.06	7.09	7.49	7.46
pCO_2_ (mmHg)	35.3	10.1	6.8	29.9	31.3
HCO_3_ (mmol/l)	23.5	7	6.6	25	23.9
BE (mmol/l)	-1,5	-26.1	-27.1	-0.2	-1.4
Na (mmol/l)	136	129	125	139	138
K (mmol/l)	3.95	4.32	4	3.76	3.76

### Follow-up

**Figure 1:**
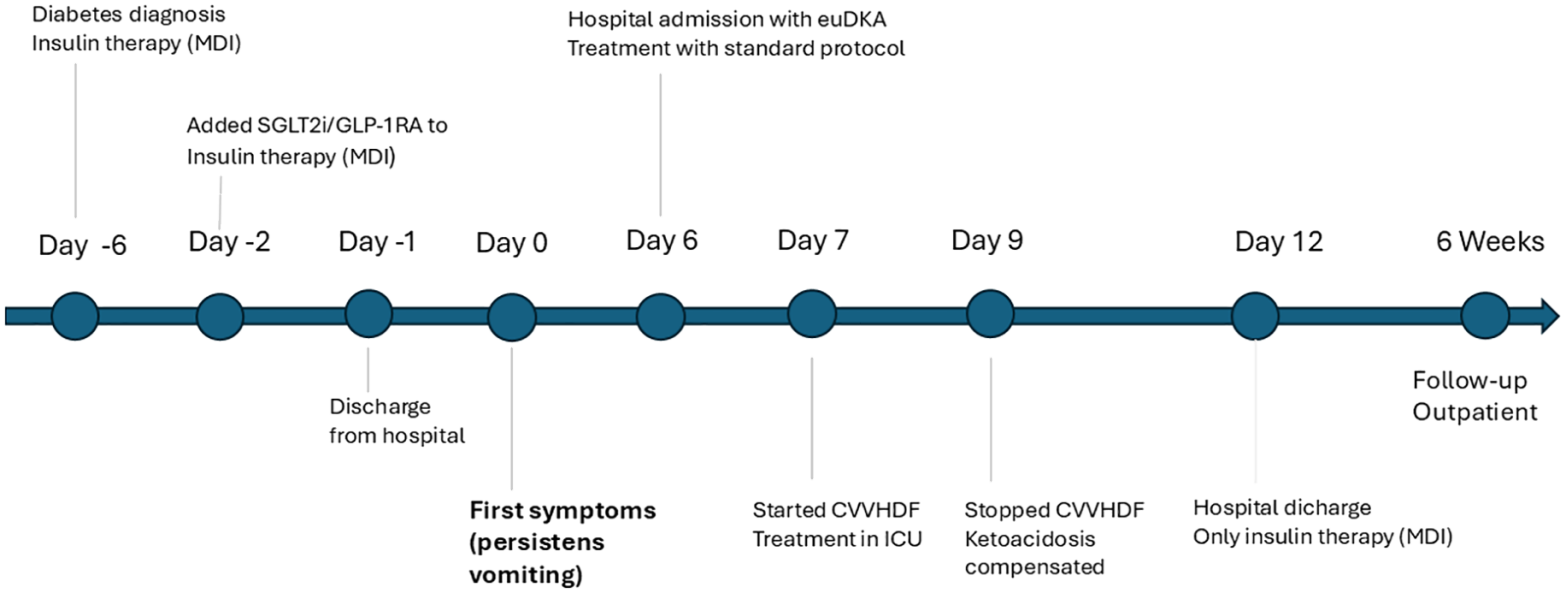
Timeline of the clinical course of the patient.

Six weeks after discharge, the patient attended a follow-up visit at the hospital outpatient clinic. He reported good functional status with no recurrence of acidosis. His body weight was 111 kg. He was treated with a basal–bolus insulin regimen (glargine 18–20 U at bedtime; prandial insulin lispro, total daily insulin dose ~50 U). Continuous glucose monitoring showed mean glucose of 171 mg/dl (9,5 mmol/l), GMI 7.4% (57 mmol/mol), CV 14.1%, with 71% time in range (70–180 mg/dl; 3,9–10 mmo/l), without hypoglycemia. He was advised to slightly increase basal insulin (20–22 U at bedtime) and scheduled for follow-up after 3 months. The timeline of the case is presented in **Figure 1**.

## Discussion

In the presented case of a newly diagnosed patient with type 2 diabetes, euDKA occurred at the very early onset after initiation of combined therapy with GLP-1RA and SGLT2i, in the absence of infection or other precipitating factors (such as alcohol overdose, pancreatitis, pregnancy), and proved refractory to standard therapy with insulin, glucose, and fluid resuscitation. A cascade of events culminated in severe, life-threatening euglycemic ketoacidosis. The described patient likely experienced adverse effects- specifically nausea and persistent vomiting-following administration of a GLP-1RA (two days after the injection) (16). The mechanism of action of GLP-1RA, including semaglutide, involves delayed gastric emptying, increased insulin sensitivity, and reduced glucagon secretion (17). These agents lead to decreased food intake, and in the presence of adverse effects such as vomiting, they may contribute to a state of starvation. This promotes lipolysis and free fatty acid oxidation through the liver, which increases the production of ketone bodies and eventually causes high anion gap metabolic acidosis. A case of euDKA has been reported in the literature in a non-diabetic female patient who was taking semaglutide for weight reduction (14). Concurrent use of SGLT2i resulted in enhanced glucosuria by blocking the SGLT2 cotransporter in the proximal renal tubule, which is responsible for 80-90% of glucose reabsorption in the kidneys (18). This led to a further decline in blood glucose levels and an increased carbohydrate deficit, as well as osmotic diuresis, which might result in volume depletion and hypovolemia (19, 20). Hypovolemia and carbohydrate deficiency lead to increased glucagon secretion, reduced endogenous insulin secretion, and elevated glucagon-to-insulin ratio, and subsequently trigger lipolysis and hepatic ketogenesis in the setting of euglycemia. Moreover, SGLT2i exert a direct effect on pancreatic alpha cells by inhibiting glucose transport into these cells, thereby further increasing glucagon secretion (21). In addition, SGLT2i enhance reabsorption of acetoacetate in the renal tubules, further contributing to elevated levels of ketone bodies in the blood (22). In the described case, glucosuria persisted for ten days following discontinuation of the SGLT2i, despite blood glucose levels below the renal threshold. This may have been due the prolonged pharmacologic activity of dapagliflozin – SGLT2i concentrations may remain relatively elevated even after drug cessation (20). Prolonged glucosuria may also be associated with dysfunction of SGLT1 and SGLT2 transporters secondary to acute proximal tubular injury caused by energy substrate deficiency (23).

We present a case of a patient with newly diagnosed type 2 diabetes who developed severe euDKA, triggered by the combination of an SGLT2i (dapagliflozin) and GLP1 RA (semaglutide). The patient received these medications in accordance with current treatment guidelines for type 2 diabetes. According to the Polish Diabetes Association, combination therapy should be particularly considered in newly diagnosed diabetes with severe hyperglycemia (HbA_1c_ > 8.5%; 69,1 mmol/mol) or very high cardiovascular risk, and the regimen should include an SGLT-2 inhibitor and/or a GLP-1 receptor agonist (15). Similarly, the ADA consensus recognizes metformin as the traditional first-line agent with stepwise intensification, but also supports initial combination therapy in patients with HbA_1c_ ≥ 1.5–2.0% above target, especially when therapies with high glycemic efficacy and proven cardiovascular or renal benefit are indicated (24). In our case autoimmune markers typical of type 1 diabetes were negative, the patient presented with newly diagnosed type 2 diabetes and markedly elevated HbA_1c_ (11.6%, 102.6 mmol/mol), BMI 32.6 kg/m^2^, hypertension, proteinuria, fulfilling criteria for early combination therapy under both sets of guidelines. However, the very early onset of euDKA after simultaneous initiation of semaglutide and dapagliflozin highlights the importance of considering staggered introduction of these agents to balance efficacy with safety.

Despite having received education and discharge instructions to check for urinary ketones in the event of nausea and vomiting, the patient ignored the alarming symptoms for an extended period, attributing them to food poisoning. In this case, the patient did not respond to standard treatment for diabetic ketoacidosis, involving intravenous fluid resuscitation, insulin therapy, and glucose infusion. Intensive care management with hemodiafiltration was required. Hemodiafiltration can be beneficial in the euDKA through the removal of ketone bodies, correction of electrolyte imbalance and volume management. Currently, several cases of treatment-resistant euDKA have been reported in the literature. In some of these cases, an additional triggering factor was identified, such as infection (25–27), the postoperative period (28, 29), or acute pancreatitis (30, 31). However, those were patients with long-lasting diabetes, in contrast to our patient, who developed eu DKA shortly after the initial diagnosis and just after the initiation of antidiabetic therapy. Consistent with our case, several reports have described the use of renal replacement therapy in the management of refractory euDKA. One case involved a patient with stage 4 chronic kidney disease treated with an SGLT-2 inhibitor and metformin, who developed euDKA with concomitant lactic acidosis. Due to resistance to standard treatment, hemodialysis was initiated for 2 days, leading to rapid improvement in acid–base status and renal function (32). Another report presented a young woman with type 2 diabetes and acute pancreatitis previously treated with dapagliflozin, who was successfully managed with continuous renal replacement therapy for 48 hours (31). Similarly, a 67-year-old woman with a 5-year history of type 2 diabetes on empagliflozin developed euDKA with acute kidney injury and standard treatment combined with bicarbonate-resistant acidosis, requiring bicarbonate hemodialysis for 2 days (33). A comparable case concerned a 48-year-old man with an 8-year history of type 2 diabetes, who developed euDKA following the introduction of dapagliflozin and discontinuation of glimepiride. Despite intensive therapy including bicarbonate supplementation, metabolic acidosis persisted and he required CVVHD (34). These reports support the role of renal replacement therapy as a valuable therapeutic option in cases of euDKA unresponsive to conventional treatment. In summary, this case reinforces the importance of recognizing euDKA as a serious adverse effect of SGLT2i, particularly when used in combination with GLP-1RA, and underscores the need for aggressive management – such as hemodiafiltration – when standard treatment protocols fail. The described case indicates that educating patients on the procedure in the event of vomiting, with control of ketonemia, is a targeted action to reduce the risk of euDKA.

## Data Availability

The original contributions presented in the study are included in the article/supplementary material. Further inquiries can be directed to the corresponding author.
